# *Bacillus subtilis* spore with surface display of paramyosin from *Clonorchis sinensis* potentializes a promising oral vaccine candidate

**DOI:** 10.1186/s13071-018-2757-0

**Published:** 2018-03-07

**Authors:** Hengchang Sun, Zhipeng Lin, Lu Zhao, Tingjin Chen, Mei Shang, Hongye Jiang, Zeli Tang, Xinyi Zhou, Mengchen Shi, Lina Zhou, Pengli Ren, Honglin Qu, Jinsi Lin, Xuerong Li, Jin Xu, Yan Huang, Xinbing Yu

**Affiliations:** 1 Department of Parasitology, Zhongshan School of Medicine, Sun Yat-sen University, 74 Zhongshan 2nd Road, Guangzhou, 510080 China; 2 Key Laboratory for Tropical Diseases Control, Sun Yat-sen University, Ministry of Education, Guangzhou, Guangdong 510080 China; 3 Provincial Engineering Technology Research Center for Diseases-vectors Control, Guangzhou, Guangdong 510080 China; 4 Department of Cell Biology and Genetics, School of Pre-clinical Medicine, Guangxi Medical University, Nanning, 530021 China

**Keywords:** *Clonorchis sinensis*, *Bacillus subtilis*, Spore, Paramyosin, Oral vaccine

## Abstract

**Background:**

Clonorchiasis caused by *Clonorchis sinensis* has become increasingly prevalent in recent years. Effective prevention strategies are urgently needed to control this food-borne infectious disease. Previous studies indicated that paramyosin of *C. sinensis* (CsPmy) is a potential vaccine candidate.

**Methods:**

We constructed a recombinant plasmid of PEB03-CotC-CsPmy, transformed it into *Bacillus subtilis* WB600 strain (*B.s*-CotC-*Cs*Pmy), and confirmed CsPmy expression on the spore surface by SDS-PAGE, Western blotting and immunofluorescence assay. The immune response and protective efficacy of the recombinant spore were investigated in BALB/c mice after intragastrical or intraperitoneal immunization. Additionally, biochemical enzyme activities in sera, the intestinal histopathology and gut microflora of spore-treated mice were investigated.

**Results:**

CsPmy was successfully expressed on the spore surface and the fusion protein on the spore surface with thermostability. Specific IgG in sera and intestinal mucus were increased after intraperitoneal and intragastrical immunization. The sIgA level in intestinal mucus, feces and bile of *B.s*-CotC-*Cs*Pmy orally treated mice were also significantly raised. Furthermore, numerous IgA-secreting cells were detected in intestinal mucosa of intragastrically immunized mice. No inflammatory injury was observed in the intestinal tissues and there was no significant difference in levels of enzyme-indicated liver function among the groups. Additionally, the diversity and abundance of gut microbiota were not changed after oral immunization. Intragastric and intraperitoneal immunization of *B.s*-CotC-CsPmy spores in mice resulted in egg reduction rates of 48.3 and 51.2% after challenge infection, respectively. Liver fibrosis degree in *B.s*-CotC-CsPmy spores treated groups was also significantly reduced.

**Conclusions:**

CsPmy expressed on the spore surface maintained its immunogenicity. Both intragastrical and intraperitoneal immunization with *B.s*-CotC-CsPmy spores induced systemic and local mucosal immune response in mice. Although both intragastric and intraperitoneal immunization elicited a similar protective effect, intragastric immunization induced stronger mucosal immune response without side effects to the liver, intestine and gut microbiota, compared with intraperitoneal immunization. Oral immunization with *B. subtilis* spore expressing CsPmy on the surface was a promising, safe and needle-free vaccination strategy against clonorchiasis.

## Background

Clonorchiasis, caused by *Clonorchis sinensis*, is one of the most common zoonoses and is of major socio-economic importance in East Asia, including China, East Russia, Korea and Vietnam [1, 2]. It is estimated that nearly 15 million people are infected and over 200 million people worldwide are under threat of *C. sinensis* infection [3, 4]. Humans or animals get infected with *C. sinensis* mainly by the consumption of raw or undercooked freshwater fish containing infective metacercariae [5]. Metacercariae excyst in the duodenum of the host, migrate into the bile duct and then develop into adult worms [6]. *Clonorchis sinensis* has been classified as one of the definite Group 1 carcinogens by the International Agency for Research on Cancer (IARC) [7]. However, it is difficult to dissuade people from eating raw fish which is deeply rooted in the epidemic area and the molecular mechanism by which the *C. sinensis* causes pathological changes is still poorly understood at present [4]. Hence, effective prevention tactics like vaccines and new antiparasitic drugs are urgently needed.

Accumulating evidence showed that immune responses in the intestinal mucosa and bile duct of hosts provoked by *C. sinensis* infection played important roles in immunotoxicity and growth inhibition on *C. sinensis*. Immune molecules presented in bile and intestinal mucosa might help to inhibit larval development and eliminate adult worms [8–10]. Therefore, oral immunization with *C. sinensis* antigen may be a convenient, inexpensive and needle-free strategy to protect humans or animals from *C. sinensis* infection. Nevertheless, oral immunization suffers from proteolysis, degradation and immune tolerance in the gastrointestinal tract, which may lead to poor immune response [11]. Choosing the ideal vehicles for the delivery of heterologous antigens to extreme environments such as the gastrointestinal tract will overcome these shortcomings.

Extensive research demonstrated that spores of *Bacillus subtilis* are an ideal platform for antigen delivery for oral vaccines [12–14]. First, protected by multiple layers of cortex proteins, the spore can survive in the presence of excessive temperature, desiccation, lytic enzymes and toxic chemicals, UV irradiation and pH [15]. In addition, utilizing the cortex coat proteins of the *B. subtilis* spore (e.g. CotB, CotC and CotG) as the anchoring protein, heterologous antigen can be stably displayed on the surface of spore [16]. Additionally, the *B. subtilis* spore is non-pathogenic and non-invasive so that is currently used in probiotics and food additives for humans and other animals [17]. Moreover, *B. subtilis* spores can be used as an immune adjuvant when administered together with purified antigenic proteins [18, 19]. In our previous studies, antigen display system based on *B. subtilis* spore was successfully established and was proved to be feasible and effective [6, 20–23].

Paramyosin, a myofibrillar protein present in numerous invertebrates including helminths, is indicated as a multifunctional molecule that related to both muscle physiological contraction and immunoregulation [24, 25]. Vaccine trials with *Schistosoma japonicum* [26, 27], *Fasciola hepatica* [28], *Schistosoma mansoni* [29] or other parasites [30–32] indicated that paramyosin was a promising vaccine candidate and elicited encouraging protective effect. In our previous study, paramyosin of *C. sinensis* (CsPmy) was confirmed to be abundantly present in the cyst wall of the metacercariae and tegument of the adult worms [33]. In addition, both the recombinant protein (rCsPmy) obtained from *Escherichia coli* expression system and the eukaryotic plasmid of pcDNA3.1(+)-CsPmy could induce strong immune responses and resulted in significant reduction rates of worm burden and eggs per gram (EPG) in vaccination trials [33].

In the present study, taking advantage of the engineering platform we constructed before, the coding sequence of CsPmy was cloned into a PEB03-CotC plasmid. The expression of fusion protein CotC-CsPmy on the surface of *B. subtilis* spore was then detected. Both the specific local and systemic immune responses of mice were analyzed after immunized with recombinant spores intraperitoneally and orally. The protective effect was also evaluated after challenging infection and the effects of recombinant spores on hepatic and intestinal functions were investigated.

## Methods

### Preparation of rCsPmy protein and antiserum

*Escherichia coli* BL21 (DE3) containing the pET-26b(+)-CsPmy plasmid was constructed in our previous study and routinely preserved in our laboratory [33]. It was induced with isopropyl-β-d-thiogalactoside (IPTG) (Sigma-Aldrich, St Louis, USA) and the inclusion bodies were dissolved in PBS containing 6 M urea. Then rCsPmy was purified with His Bind Purification kit (Novagen, Darmstadt, Germany), eluted with gradient imidazole (Sigma-Aldrich) solution. Purified rCsPmy were renatured, analyzed by sodium dodecyl sulfate-polyacrylamide gel electrophoresis (SDS-PAGE), as described before [33]. Then rCsPmy was emulsified with complete Freund’s adjuvant and subcutaneously injected to SD rats. Each animal was given 200 μg recombinant protein for the first injection, and 100 μg recombinant protein emulsified with incomplete Freund’s adjuvant was given for the next two boosters at a 2-week interval. 2 weeks after the last injection, the rat sera were collected and stored at -80 °C after determination of antibody titer by ELISA as described before [33].

### Construction of PEB03-CotC-CsPmy

To display CsPmy on the surface of *B. subtilis* (WB600) spores, the full-length coding sequence of CsPmy (GenBank: EF071860.1) was connected to the 3' end of the coding sequence of CotC, a component of spore coat proteins, to construct fusion gene of CotC-CsPmy. Briefly, PEB03-CotC, a plasmid previously constructed and routinely stored in our laboratory, was linearized by using *Hind*III and *Sac*I as restriction sites. The complete coding sequence (CDS) of CsPmy was amplified from the cDNA of *C. sinensis* adult with specific primers. The sequence of the forward primer is 5'-CAT AAA AAA CAC TAC AAG CTT ATG AGT CAC GAG TCG GAA TCA CA-3' (underlined are homologous sequences of PEB03-CotC), the sequence of the reverse primer is 5'-AGT GGC AAA GTG CTA GAG CTC TTA CAT CAT GCT CGT CGC GC-3' (underlined are homologous sequences of PEB03-CotC). The amplified products were recovered by a gel extraction kit (TIANGEN, Beijing, China) and then subcloned into a linearized PEB03-CotC vector with a ClonExpress One Step Cloning Kit (Vazyme, Nanjing, China) in strict accordance with the manufacturer’s instructions. The recombinant plasmid PEB03-CotC-CsPmy was employed as template for PCR amplification of CotC, CsPmy or CotC-CsPmy gene fragments for verification and was further confirmed by DNA sequencing. Finally, the recombinant plasmid was transformed into *B. subtilis* WB600 strain (routinely preserved in our laboratory) for fusion protein expression [34]. The transformed strain of *B.s*-CotC-CsPmy was obtained. The PEB03-CotC plasmid was also transformed into WB600 (*B.s*-CotC) and used as a control.

### Preparation of recombinant spores

*B.s*-CotC and *B.s*-CotC-CsPmy were grown in Difco Sporulation Medium (DSM) to induce sporulation by the exhaustion method as described before [35, 36]. Sporulation medium was harvested 24 h after the initiation of sporulation and treated with lysozyme to break residual sporangial cells. Then spores were successively washed with 1 M NaCl, 1 M KCl, and washed two times with distilled water. Phenylmethylsulfonyl fluoride (PMSF, 1 mM) (Sigma-Aldrich) was added in each washing step to inhibit proteolysis. After the final wash, spores were put into a 65 °C water bath for 1 h to kill residual vegetative cells. The number of spores was calculated with a Bürker chamber under an optical microscope. Spore samples were used immediately or kept at -80 °C until use.

### SDS-PAGE, Western blotting, thermostability analysis of CsPmy on spore surface

SDS-DTT extraction buffer (0.5% SDS, 0.1 M DTT, 0.1 M NaCl) was used for the extraction of spore coat proteins as previously described in detail [36]. Then the extracted proteins were subjected to 12% SDS- PAGE and visualized by Coomassie brilliant blue G-250 staining.

Coat proteins were also transferred onto a polyvinylidene fluoride (PVDF) membrane (Millipore, Billerica, USA) after SDS-PAGE. After overnight blocking with 5% skim milk in phosphate buffered saline (PBS)-Tween (PBST), the membrane was incubated with rat-anti CsPmy sera (1:2000 diluted with PBST) for 2 h at room temperature (RT). Followed by 5 times washing with PBST, the membrane was incubated with horseradish peroxidase (HRP)-conjugated rabbit anti-rat antibody (1:5000 diluted with PBST, Sigma-Aldrich) and finally visualized by the chemiluminescence method.

For thermostability analysis, an equal volume (0.1 ml) of spore suspension was packaged in Eppendorf tubes and stored at RT, 4 °C, -40 °C, or -80 °C (three replicates per temperature condition) for 3 months. Spores were finally collected for 12% SDS-PAGE analysis.

### Immunofluorescence analysis

Spores were washed three times with distilled water and fixed on slides as described in the previous report [37]. The slides were blocked with normal goat serum overnight at 4 °C followed by incubation with anti-CsPmy sera (1:200 diluted with PBST) for 1.5 h at RT. Naïve rat serum at the same dilution was used as a control. After washing thoroughly, Cy3-labeled goat anti-rat IgG (Invitrogen, Carlsbad, USA, 1:400 in PBST) was employed to visualize the reaction. Finally, samples were detected and photographed under a fluorescent microscope (Leica DFC500 Digital Camera, Barnack, German) in dark.

### Immunization of mice and sample collection

**Fig. 1:**
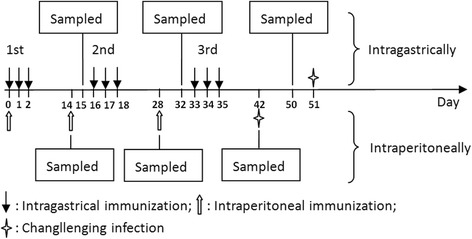
Schematic of the immunization and sampling programs. For intraperitoneal immunization, mice were intraperitoneally injected with PBS, *B.s*-CotC spores, *B.s*-CotC-CsPmy spores for three times. For oral immunization, mice were intragastrically immunized with PBS, *B.s*-CotC spores, and *B.s*-CotC-CsPmy spores for three times, with continuous three days per time. Serum, feces, intestinal mucus, bile samples were collected on day 15, 32 and 50. For intraperitoneally immunized mice, samples were collected on day 14, 28 and 42. Immunized mice were challenging infected with *C. sinensis* metacercaria two weeks after the final immunization

To investigate the immunogenicity induced by *B.s*-CotC-CsPmy spores in different immunization routes, we carried out the vaccine experiment in BALB/c mice. The immunization and sample collection programs are described in Fig. 1.

In detail, for intraperitoneal immunization, mice were equally divided into three groups and were intraperitoneally injected with 1.0 × 10^11^ *B.s*-CotC-CsPmy spores (*n* = 20), *B.s*-CotC spores (*n* = 20) and PBS (*n* = 20) in a volume of 0.1 ml on days 0, 14 and 28. Five mice were sacrificed and serum, intestinal mucus, bile, and feces were collected on day 14, 28, 42. Fecal pellet (0.1 g) was suspended in PBS with bull serum albumin (BSA, 1%) and phenylmethanesulfonyl fluoride (PMSF) (1 mM), incubated at 4 °C overnight, and then centrifuged. The supernatant was stored at -20 °C prior to ELISA.

For intragastric immunization, mice were immunized according to the immune procedure used in previous studies [6, 21, 22, 37]. In detail, BALB/c mice (female, 6–8 weeks-old) were randomly divided into three groups as *B.s-*CotC-CsPmy (*n* = 20), *B.s-*CotC (*n* = 20) and PBS (*n* = 20). Mice in groups of *B.s*-CotC-CsPmy and *B.s-*CotC were, respectively, intragastrically administrated with 1.0×10^11^ recombinant spores in 0.1 ml of PBS by intragastric lavage on day 0, 1, 2, 16, 17, 18, 33, 34 and 35. Mice in the PBS group were given the same volume of PBS. Five mice in each group were sacrificed by anesthesia on day 15, 32 and 50. The serum, bile, intestinal mucus and feces were collected and preserved in -20 °C as described elsewhere [23]. On day 50, duodenum, jejunum, and ileum of the mice were sterilely isolated. One part of the intestine (2 cm in length) was cut then immersed in 4% paraformaldehyde.

### Detection of CsPmy-specific antibodies by ELISA

ELISA plates were coated with 50 μl per well of purified rCsPmy protein (5 μg/ml) in coating buffer (0.05 M carbonate-bicarbonate, pH 9.6) and blocked with PBST containing 5% skimmed milk at 37 °C for 2 h. After washing three times with PBST, the plates were incubated with serum (1:200 dilutions) for 1 h at 37 °C. Subsequently, HRP-conjugated rabbit anti-mice IgG (1:8000 dilutions, Proteintech Group, Chicago, USA), IgG1 or IgG2a (1:2000 dilutions, Bethyl, Montgomery, USA) were added as secondary antibodies. Plates were incubated for 1 more hour at 37 °C and then reacted with the substrate TMB (BD, Franklin Lakes, USA). After 10 min incubation in dark, reactions were stopped by adding 50 μl of 2 M H_2_SO_4_ and the absorbance was measured at 450 nm by a microplate reader.

ELISA detection of sIgA in intestinal mucus, bile or feces was done with the similar method as above. After blocking with PBST containing 5% skim milk, intestinal mucus (1:50 dilutions), bile (1:100 dilutions), or extracted fecal supernatant (1:10 dilutions) were subjected to the wells. Finally, sIgA levels were detected using HRP-goat anti-mice IgA antibody (1:2000 dilution).

### Immunohistochemistry detection of IgA-secreting cells

Fixed intestinal fragments of mice were embedded in paraffin and sliced into 5 μm sections. The sections were deparaffinized with dimethylbenzene, rehydrated with graded ethanol, and then blocked with normal goat serum at RT for 2 h. Subsequently, the slides were incubated with goat anti-mice IgA (1:250 dilutions, Novus, St. Louis, USA) at 4 °C overnight. Following the washing procedures, the slides were treated with HRP-Protein A (1:4000 dilutions, GenScript, New Jersey, USA) at 37 °C for 1 h. The slides were developed color with 3, 3-diaminobenzidine (DAB) at RT under darkness for 12 min and stopped with distilled water. Finally, cell nuclei were stained with hematoxylin. Positive cells were stained dark brown. Thirty microscopic fields of each sample were randomly captured. The images were analyzed by ImagePro Plus software (Media Cybernetics, Roper, USA). The number of IgA-secreting cells was indicated by integrated optical density (IOD). The sizes of the area of interest (AOI) were the same.

### Challenging infection and evaluation of protective efficacy

Metacercariae of *C. sinensis* were collected from infected *Pseudorasbora parva* captured in Shuangfeng County, Hunan. The fish muscle was cut into pieces and digested using artificial gastric juice for an hour at 37 °C; then metacercariae were identified and collected under a dissecting microscope as described before [38]. Two weeks after the final booster immunization, the mice were given a challenging infection. Briefly, 40 metacercariae of *C. sinensis* were orally gavaged to mice by a gavage needle. Five weeks after the challenging infection, feces were collected to calculate the EPG according to Kato-Katz method. Stool samples from mice were applied to prepare triplicate Kato-Katz thick smears using 41.7 mg templates. The smears were then examined under an optical microscope by experienced technicians and the number of *C. sinensis* eggs was counted and recorded [39, 40]. The protective efficacy was evaluated by egg reduction rate (%) as per the published method [33]. Egg reduction rate (%) = (1-average EPG of experimental group/average EPG of the control group) × 100%. The mice were sacrificed 8 weeks post-infection, and then livers were carefully isolated and were fixed with 4% paraformaldehyde. The livers were sliced into 5 μm sections. After Masson trichrome staining, the sections were examined using a microscope. The Ishak fibrosis score was applied to estimate the severity of liver fibrosis as described elsewhere [41].

### Histology staining

For pathological observations, fixed intestinal fragments of mice (*n* = 5) in each group were sliced into sections (5 μm thick), and turned into the hematoxylin and eosin (H&E) staining and viewed under a light microscope (Carl Zeiss, Jena, Germany).

### Detection of biochemical indices

The sera of mice (*n* = 5) in each group were collected at day 50. The glutamic pyruvic transaminase/alanine aminotransferase (GPT/ALT) and glutamic oxaloacetic transaminase/aspartate aminotransferase (GOT/AST) were measured by using the Alanine aminotransferase Assay Kit and the Aspartate aminotransferase Assay Kit (Jiancheng, Nanjing, China), respectively.

### Intestinal microbiota analysis

One month after the last immunization, feces (1 g) of mice in the PBS group (*n* = 5) and the *B.s*-CotC-CsPmy group (*n* = 5) were collected. Total genome DNA of feces were extracted using a Fecal Genomic DNA Extraction Kit (Cwbiotech, Beijing, China). *16S* rRNA genes of distinct regions (16SV4) were amplified using specific primers and were sequenced on an IlluminaHiSeq2500 platform (Illumina, San Diego, USA) using well-established programs. Sequence analyses were performed by Uparse software (Uparse v.7.0.100, http://drive5.com/uparse/) [42]. Sequences with ≥ 97% similarity were assigned to the same operational taxonomic units (OTUs). The representative sequence for each OTU was screened for further annotation. For each representative sequence, the GreenGene Database (http://greengenes.lbl.gov/) [43] was used based on the RDP classifier (Version 2.2, http://sourceforge.net/projects/rd p-classifier) [44] algorithm to annotate taxonomic information. Alpha diversity was applied in analyzing the complexity of species diversity for a sample through different indices: the Chao1 and abundance-based coverage estimator (ACE) indices were selected to identify community richness, while the Shannon and Simpson indices were used to identifying community diversity. All these indices in our samples were calculated with QIIME (Version 1.7.0) and displayed with R software (Version 2.15.3). Alpha diversity indices of the PBS group and the spores treated group were compared to evaluate the changes in the composition of intestinal microbiota.

### Statistical analysis

Experimental data were expressed as the mean ± standard deviation (SD) values. Student’s t-test was performed to determine significant differences between groups using SPSS software 13.0. Where *P*-values < 0.05, differences between groups were considered as statistically significant.

## Results

### Construction of recombinant plasmid of PEB03-CotC-CsPmy and antibody titer of CsPmy antiserum

**Fig. 2:**
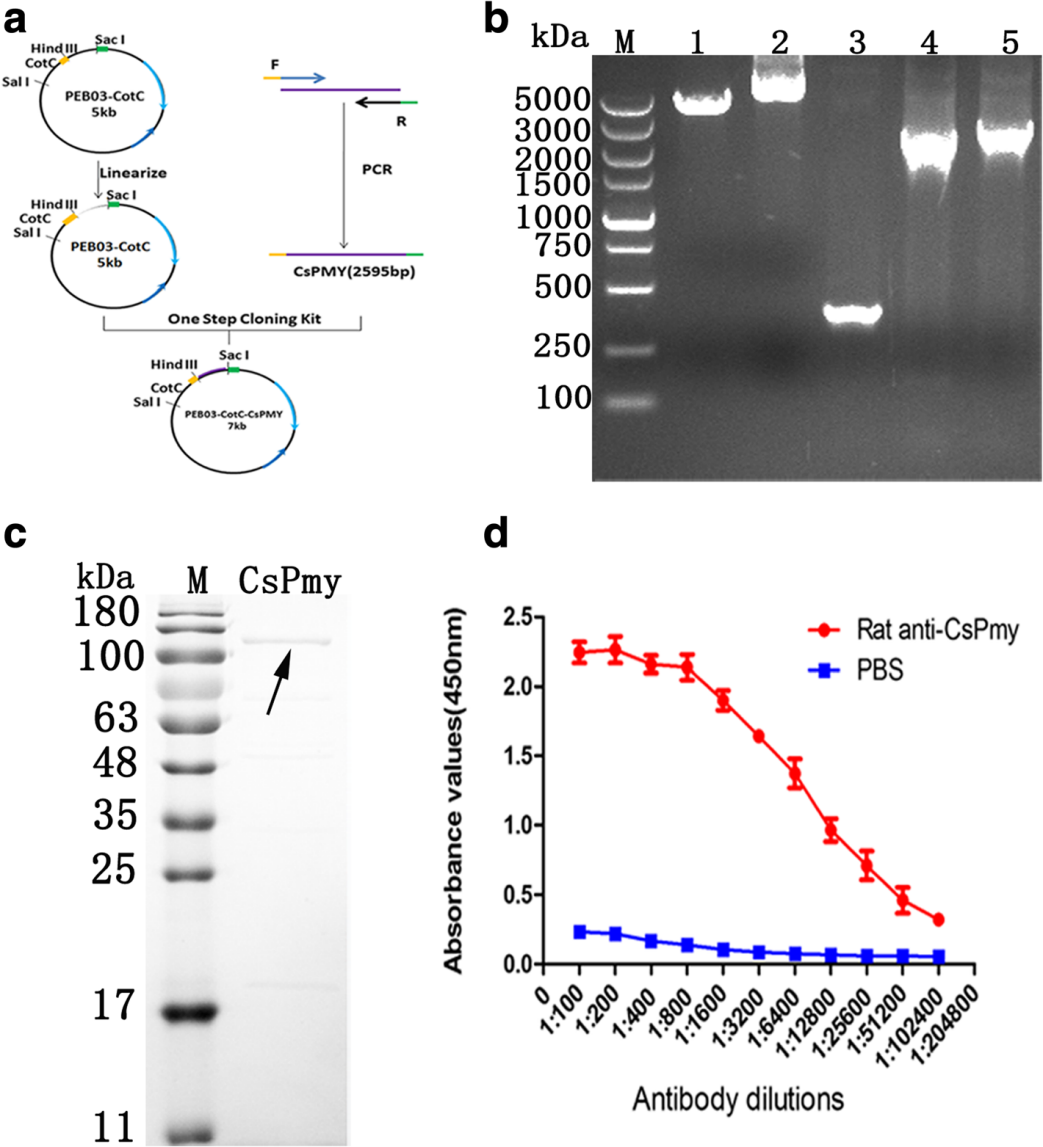
Construction of recombinant PEB03-CotC-CsPmy plasmid and preparation of rCsPmy and antibody titer analysis. **a** Schematic representation of genetic engineering using PEB03-CotC. **b** The full-length coding sequence of CsPmy was cloned into a PEB03-CotC plasmid and was confirmed by PCR with recombinant PEB03-CotC-CsPmy plasmid as template. Lane M: DNA markers; Lane 1: PEB03-CotC plasmid; Lane 2: recombinant PEB03-CotC-CsPmy plasmid; Lane 3: CotC gene. Lane 4: CsPmy gene; Lane 5: CotC-CsPmy gene. **c** SDS-PAGE analysis of purified recombinant CsPmy. *Abbreviations*: M, protein marker; CsPmy, recombinant CsPmy protein. **d** The antibody titer of IgG in serum of CsPmy immunized rat

The recombinant plasmid PEB03-CotC-CsPmy was constructed under the schematic representation shown in Fig. 2a. The full-length coding sequence of CsPmy (2595 bp) was ligated to the 3' end of CotC of PEB03-CotC vector. CotC, CsPmy, CotC-CsPmy gene fragments were successfully amplified by PCR with the recombinant plasmid as a template (Fig. 2b). The PEB03-CotC-CsPmy plasmid was also confirmed by DNA sequencing and transformed into *B. subtilis* WB600 strain. The recombinant CsPmy was expressed as inclusion bodies after induction and the molecular mass was about 100 kDa (Fig. 2c). The antibody titer reached 1:102400 two weeks after the third immunization (Fig. 2d).

### Expression identification and thermostability analysis of CotC-CsPmy fusion protein

**Fig. 3:**
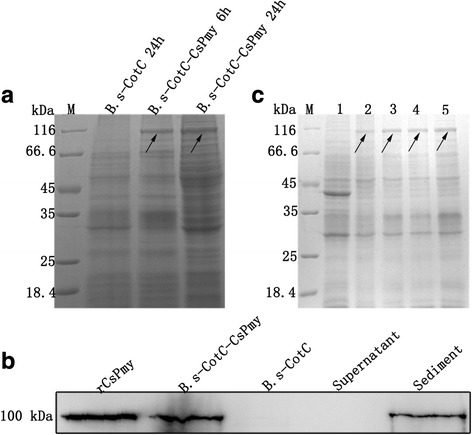
Expression identification and thermostability analysis of CotC-CsPmy on *B. subtilis* spores. **a** SDS-PAGE analysis of the fusion expression on *B. subtilis* spores in different sporulation times. The arrows indicated the possible expression band. The molecular mass of fusion protein was approximately 108.8 kDa. **b** Identification of CotC-CsPmy fusion protein with Western blotting by using rat anti-rCsPmy serum. There was no corresponding band in lanes of *B.s*-CotC spores and the supernatant of *B.s-*CotC-CsPmy spores. **c** Thermostability analysis of fusion protein on *B. subtilis* spores. An equal quantity of spore was packaged in Eppendorf tubes and stored at RT, 4 °C, -40 °C, or -80 °C for 3 months, and then analyzed by using 12% SDS-PAGE. Lane M: Molecular weight marker; Lane 1: *B.s*-CotC spores stored at -80 °C; Lanes 2–5: *B.s*-CotC-CsPmy stored at room temperature, 4 °C, -40 °C or -80 °C, respectively. The fusion protein bands are indicated by arrows

Recombinant plasmid PEB03-CotC-CsPmy was transformed into *B. subtilis* WB600 strain and induced for sporulation in DSM medium. The molecular weight of the fusion protein of CotC-CsPmy was approximately 108.8 kDa, corresponding to the molecular mass of CsPmy (100 kDa) plus CotC (8.8 kDa). The band of the fusion protein was detected 6 and 24 h after the induction, while no corresponding band was observed in the *B.s*-CotC spores after 24 h induction (Fig. 3a). Western blotting showed that CotC-CsPmy protein could be probed in the sediment of *B.s*-CotC-CsPmy spores, while no band was observed in supernatant or sediment of *B.s*-CotC spores (Fig. 3b). The thermostability of fusion protein on spore coat was analyzed by SDS-PAGE. As shown in Fig. 3c, spores were stored at RT, 4 °C, -40 °C, or -80 °C for 3 months. The fusion protein band was still able to be detected. The bands at different temperatures were the same.

### Immunofluorescence analysis of CsPmy expressed on the spore surface

**Fig. 4:**
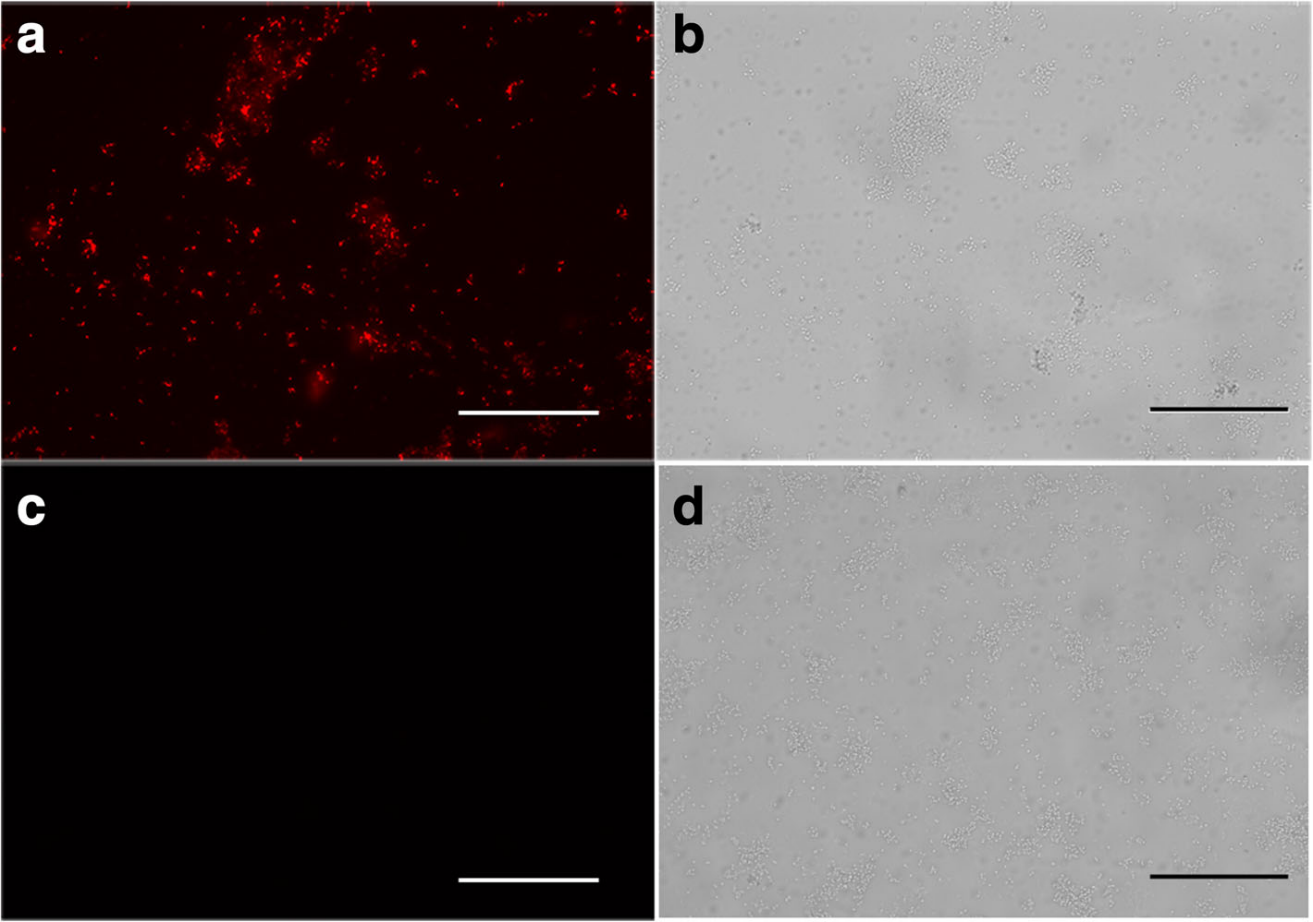
Immunofluorescence analysis of CsPmy expressed on the spore surface. Spores were fixed on slides, incubated with anti-rCsPmy rat serum and cy3-labeled goat anti-rat IgG. Panels **a** and **c** were visualized under fluorescent light and panels **b** and **d** were observed under bright field. Panels **a** and **c** are *B.s*-CotC-CsPmy spores and panels **c** and **d** are *B.s*-CotC spores. All images were magnified at 400×. *Scale-bars*: 50 μm

Immunofluorescence was employed to further confirm the expression of CsPmy on the surface of the spore coat. Using the rat anti-rCsPmy serum as the primary antibody and followed by Cy3-labelled goat anti-rat IgG as the secondary antibody, red fluorescence was clearly distributed around the surface of *B.s*-CotC-CsPmy spores (Fig. 4a, b). However, no fluorescence was observed on the *B.s*-CotC spores surface (Fig. 4c, d).

### Specific IgG and isotypes in serum of immunized mice

**Fig. 5:**
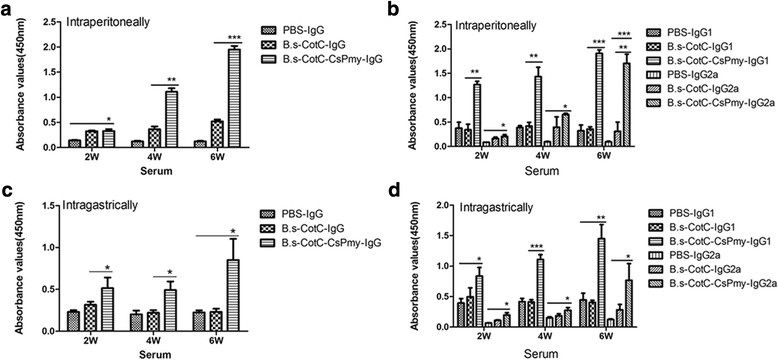
Detection of specific IgG and isotypes in sera from spore treated mice by ELISA assay. IgG levels in sera triggered by intraperitoneally (**a**) and intragastrically (**c**) immunized with recombinant spores. IgG1 and IgG2a levels in sera triggered by intraperitoneally (**b**) and intragastrically (**d**) immunized with recombinant spores. Data was expressed as the mean ± SD. Statistical significance was analyzed by the Student’s t-test (*P < 0.05, **P < 0.01, ***P < 0.001)

IgG levels in serum rose two weeks after the first injection with recombinant spores and consistently elevated during immunization. In the *B.s*-CotC-CsPmy-injected group, specific IgG levels of mice was significantly higher than those in the *B.s*-CotC group or the PBS group after week 2 (*t*_(8)_ = 4.937, *P* = 0.0178), while no significant difference was detected between the *B.s*-CotC group and the PBS group (Fig. 5a). Specific IgG1 levels in serum of mice in the *B.s*-CotC-CsPmy-injected group was also remarkably elevated in each time point (*t*_(8)_ = 6.416, *P* = 0.003) (Fig. 5b). The IgG2a levels were lower than IgG1 levels but were significantly boosted from week 2 to week 6 in the *B.s*-CotC-CsPmy group (*t*_(8)_ = 3.345, *P* = 0.0287) (Fig. 5b).

The levels of specific IgG in serum from mice of the *B.s*-CotC-CsPmy oral immunized group were higher compared with those in mice orally treated with the *B.s*-CotC group and the PBS group (Fig. 5c). Similarly, in the *B.s*-CotC-CsPmy spores group, specific IgG1 (*t*_(8)_ = 2.816, *P* = 0.0226) and IgG2a levels (*t*_(8)_ = 3.330, *P* = 0.0104) were also significantly elevated compared with the control groups from week 2 to week 6 (Fig. 5d).

### Analysis of local mucosal antibodies

**Fig. 6:**
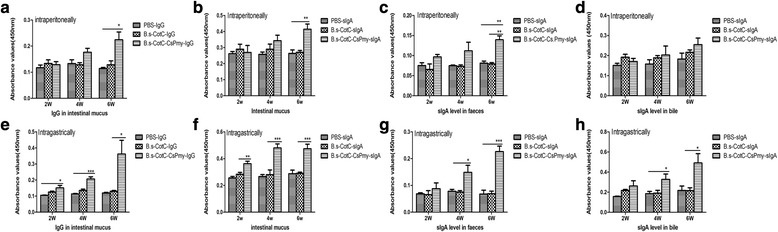
Antibody levels in feces, intestinal mucus, and bile from mice immunized with recombinant spores. CsPmy-specific IgG levels in intestinal mucus (**a**, **e**); CsPmy-specific sIgA levels in intestinal mucus (**b**, **f**), feces (**c**, **g**), and bile (**d**, **h**) from immunized mice. **a-d** samples from intraperitoneally immunized mice, **e**-**h** samples from intragastrically immunized mice. Data are presented as the mean ± SD. The Student’s t-test was applied to analyze statistical significance among the groups (*P < 0.05, **P < 0.01, ***P < 0.001)

Antibody levels were analyzed by ELISA and the results showed that in intraperitoneally immunized mice the IgG level in intestinal mucus was significantly upraised at week 6 (*t*_(8)_ = 3.648, *P* = 0.0065 ) (Fig. 6a). Additionally, sIgA levels in intestinal mucus (*t*_(8)_ = 3.828, *P* = 0.005) and in feces (*t*_(8)_ = 5.828, *P* = 0.0060) were also significantly different compared with the control groups (Fig. 6b, c). However, the sIgA level in the bile of intraperitoneally immunized mice was not significantly changed (Fig. 6d).

After intragastric immunization, compared with the control groups, the specific IgG level in intestinal mucus in the *B.s*-CotC-CsPmy group was significantly elevated in week 2 (*t*_(8)_ = 2.825, *P* = 0.0223), and was dramatically raised in week 4 and week 6 (Fig. 6e). Similarly, sIgA levels in the intestinal mucus (*t*_(8)_ = 4.303, *P* = 0.0026), feces (*t*_(8)_ = 6.303, *P* = 0.0032) and bile (*t*_(8)_ = 3.034, *P* = 0.0386) of mice in the *B.s*-CotC-CsPmy spores group was also remarkably increased in week 4 and week 6, respectively (Fig. 6f-h).

### Immunohistochemistry assay of IgA-secreting cells in intestinal epithelium

**Fig. 7:**
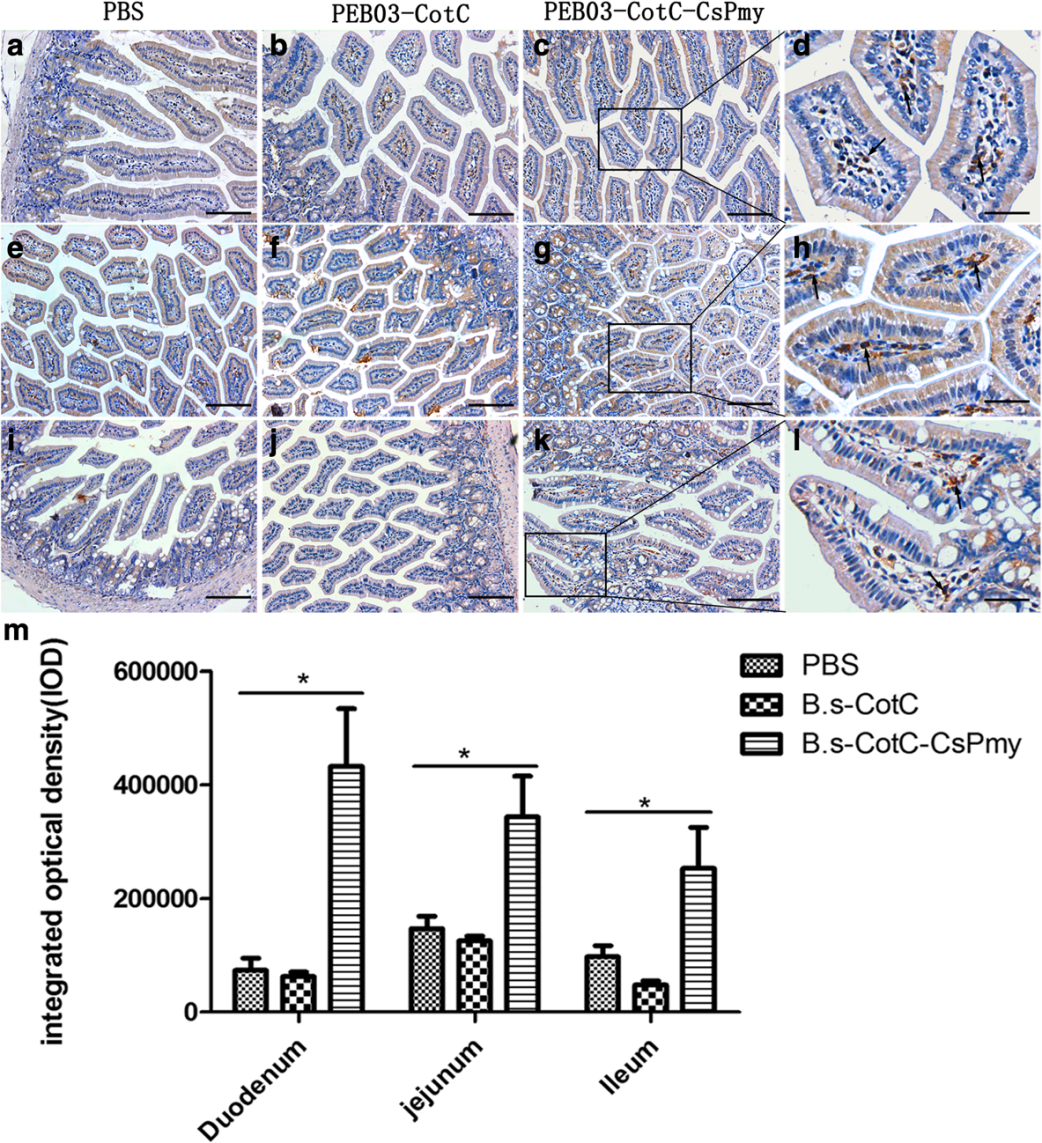
Immunohistochemistry analysis of IgA-secreting cells in the intestinal epithelium of intragastrically treated mice. IgA-secreting cells were stained with dark brown. Images of the intestinal epithelium, representative fields of duodenum (**a**-**d**), jejunum (**e**-**h**) and ileum (**i**-**l**) are shown for PBS group (**a**, **e**, **i**), *B.s*-CotC spores group (**b**, **f**, **j**) and *B.s*-CotC-CsPmy spore group (**c**, **d**, **g**, **h**, **k**, **l**). **m** Integrated option density (IOD) of IgA-secreting cells (*P < 0.05). Images in panels **a**, **b**, **c**, **e**, **f**, **g**, **i**, **j** and **k** are taken at a magnification of 100×, those in panels **d**, **h** and **l**) are taken at a magnification of 200×. The arrows indicate the IgA-secreting cells. *Scale-bars*: **a**-**c**, **e**-**g**, **i**-**k**, 100 μm; **d**, **h**, **l**, 50 μm

Using goat anti-mice IgA and HRP-protein A, the sIgA cells were stained with dark brown. There were many positive cells distributed around enteraden of the duodenum, jejunum, and ileum in the *B.s*-CotC-CsPmy group (Fig. 7c, d, g, h, k, l). However, fewer positive cells were seen in the PBS or *B.s*-CotC group (Fig. 7a, b, e, f, i, j). IgA-secreting cells were indicated by IOD using ImagePro Plus software. In the *B.s*-CotC-CsPmy group, IODs in duodenum, jejunum, and ileum were all statistically higher than those of the control groups (PBS group, *B.s*-CotC group) (*t*_(8)_ = 3.018, *P* = 0.0288), while there was no difference between the *B.s*-CotC and PBS groups. Additionally, the IOD of the duodenum was the highest in the *B.s*-CotC-CsPmy group, followed by that of jejunum. The IOD of the ileum is the lowest (Fig. 7m).

### Protective efficacy evaluation

Feces of mice in each group were collected 30 days after the challenging infection. *Clonorchis sinensis* eggs were counted and presented as eggs per gram of stool. Data are shown as the mean ± standard deviation (SD). The average EPG of the *B.s*-CotC-CsPmy spores group was 39.60 ± 3.945 (intragastrically) and 37.40 ± 3.647 (intraperitoneally). The egg reduction rates were 48.3 and 51.2%, respectively, compared with the control groups (Table 1).

**Table 1:** Protective effect of spores immunized by different routes. Protective effect was evaluated by comparing EPG between groups. Results for analysis represented mean ± SD and the EPG in groups were compared using Student’s t-test. Adult worm was not found in each group, so the worm burden was not given

Group	EPG	Egg reduction rate (%)
PBS (*n* = 5)	76.60 ± 7.427	–
*B.s*-CotC (n *=* 5)	74.80 ± 8.194	–
Oral (n *=* 5)^a^	39.60 ± 3.945^**^	48.30
Injected (*n* = 5)^b^	37.40 ± 3.647^**^	51.20

^a^Orally immunized mice

^b^Intraperitoneally injected mice

**P < 0.01 (compared to corresponding control)

### Histopathological analysis and enzyme indicators of liver function

**Fig. 8:**
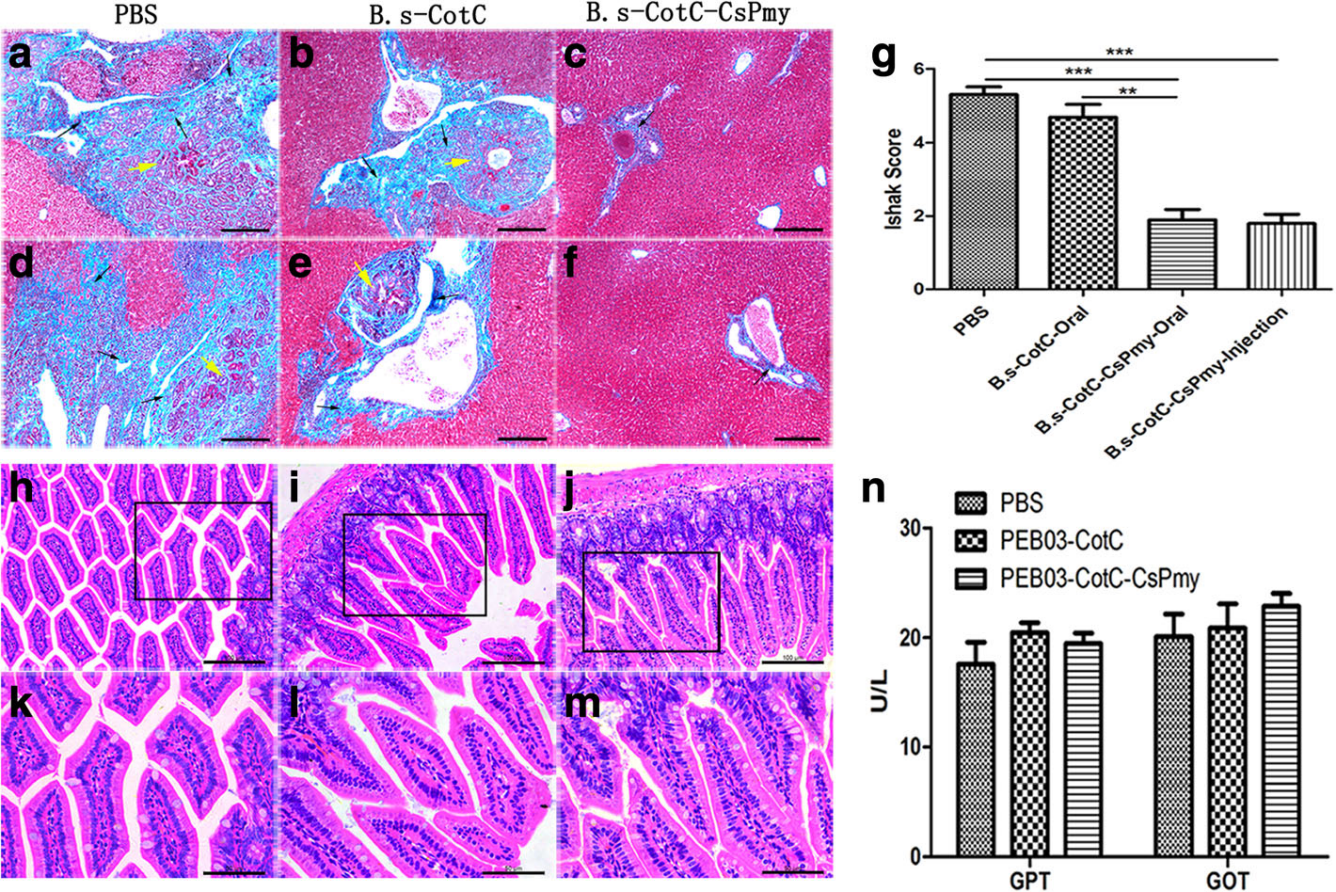
Histopathological analysis of liver and intestine and levels of enzyme indicators. **a**-**f** Liver slides by using Masson’s trichrome staining. Collagen fibers were dyed blue. **a**-**c** Representative fields of liver tissues from mice intraperitoneally injected with PBS, *B.s*-CotC spores, *B.s*-CotC-CsPmy spores. **d-f** Representative fields of liver tissues from mice intragastrically immunized with PBS, *B.s*-CotC spores and *B.s*-CotC-CsPmy spores, respectively. The yellow arrows indicate adenomatoid hyperplasia, the black arrows indicate the collagen deposition around the bile duct. **g** Statistical analysis of Ishak scores in liver sections from each group (**P < 0.01, ***P < 0.001). **h-m** Histological detection of the intestines from orally immunized mice by H&E staining: **h**, **k** PBS group; **i**, **l** *B.s*-CotC group; **j**, **m** *B.s*-CotC-CsPmy group (**h**-**j**, 100×; **k**-**m**, 200×). **n** The activities of GPT and GOT in sera of intragastrically immunized mice. *Scale-bars*: **a**-**f**, 50 μm; **h**-**m**, 100 μm

Livers of mice were sliced and turned to Masson staining 8 weeks after the challenge infection. There was an abundant collagen deposition around bile ducts and in hepatic parenchyma in the PBS group or *B.s*-CotC group (Fig. 8a-f). Additionally, bile duct hyperplasia was observed in these groups. However, the collagen deposition and hyperplasia phenomenon were much lighter in the *B.s*-CotC-CsPmy group. Ishak scores demonstrated that intragastrical treatment with *B.s*-CotC-CsPmy spores dominantly reduced the degree of hepatic fibrosis in challenge infected mice (*t*_(18)_ = 9.725, *P* = 0.0001) (Fig. 8g).

No inflammatory injury or other histopathological changes were detected in H&E stained intestines of mice in orally treated groups (Fig. 8h-m). In addition, there was no statistical difference in GOT or GPT activities in serum among the groups (Fig. 8n).

### Intestinal microbiota analysis

**Fig. 9:**
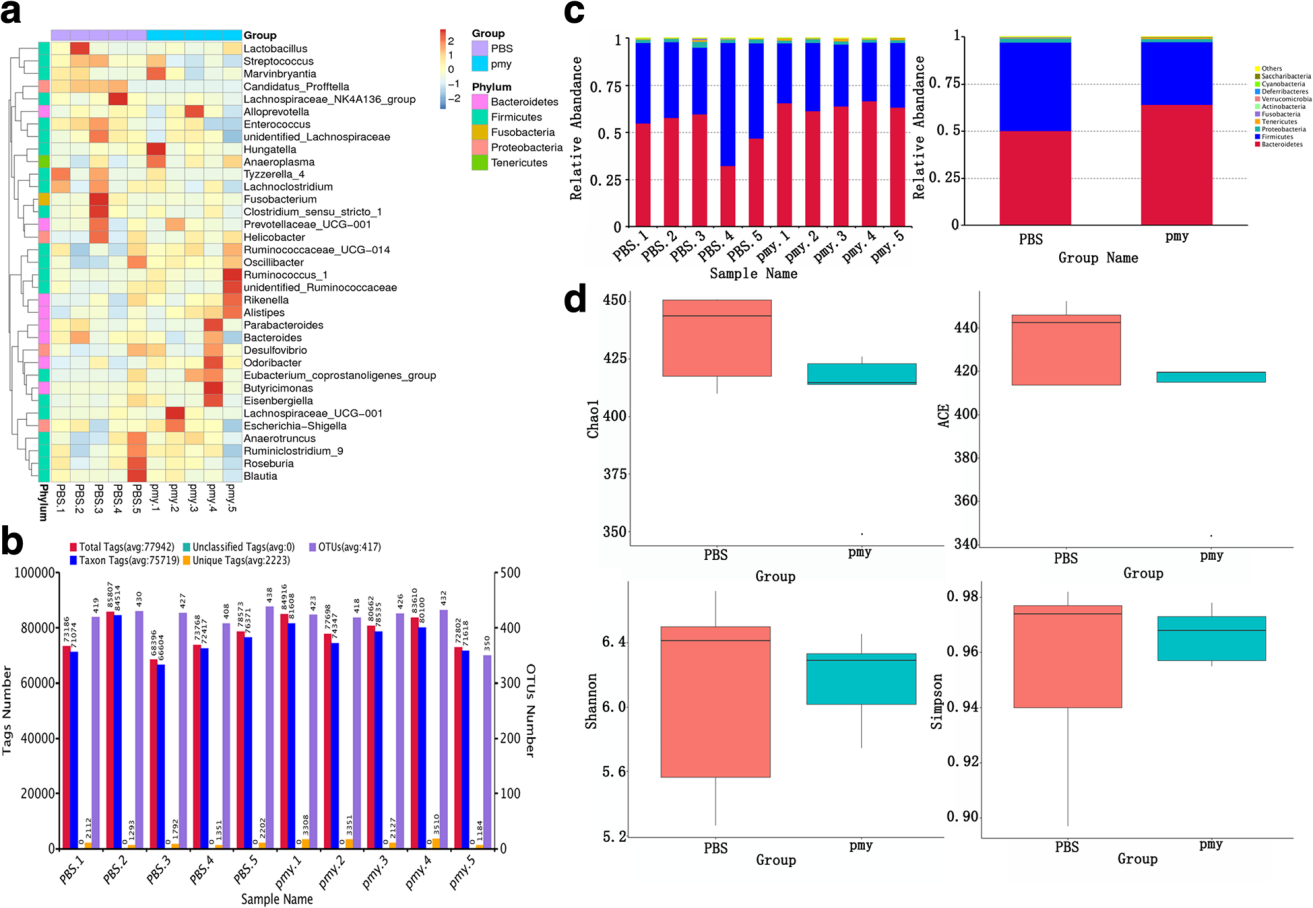
Analysis of gut microflora by *16S* rRNA sequencing. **a** Heatmap of species abundance and clustering at the genus level. **b** OTUs clustering and annotation statistics of each sample. **c** The top ten species with the highest abundance at Phylum level in each group. The relatively abundant species were plotted in order to visualize the relative abundance of species and their proportions. **d** Analysis of the diversity indices between PBS and spore treated groups. Chao1 and ACE indices were chosen to identify community richness. Shannon and Simpson indices were selected to identify community diversity. The Student's t-test and Wilcox rank sum test showed that there was no obvious difference in all the indices between the two groups. *Abbreviations*: PBS, PBS-treated group; pmy, *B.s*-CotC-CsPmy spores-treated group

No obvious difference in the species abundance and clustering at the genus level were detected in the PBS group and spore group (Fig. 9a). An average of 425 and 410 operational taxonomic units (OTUs) was identified in the PBS group and *B.s*-CotC-CsPmy orally treated group, respectively (Fig. 9b). At the phylum level, Bacteroidetes, Firmicutes and Proteobacteria were the dominant phyla (the total relative abundance > 95%) in both the PBS- and spores-treated groups (Fig. 9c). There was no significant difference in Chao1, ACE, Shannon, and Simpson indices representing the bacterial community richness and community diversity (Fig. 9d).

## Discussion

In the present study, CsPmy was successfully displayed on the surface of *B. subtilis* spores using CotC as a fusion partner. An immune reaction was elicited in mice administrated with recombinant spores (*B.s*-CotC-CsPmy). When intragastrically administrated with recombinant spores, the specific IgG levels in sera and the intestinal mucus were remarkably increased, as well as the sIgA levels in bile and intestinal mucus. Numerous IgA-secreting cells were also detected in the intestinal mucosa of mice orally treated with *B.s*-CotC-CsPmy spores. In addition, the GOT and GPT activities in sera of mice in each group showed no statistically significant difference. Additionally, no inflammatory damage was observed in the intestinal tissue and no obvious difference in the species abundance and clustering at the genus level between the PBS group and the spore group by intestinal microbiota analysis. More encouragingly, 48.3% (oral administration) and 51.2% (intraperitoneal administration) of egg reduction rates were elicited by spore immunization. Furthermore, compared with control groups, the collagen deposition in liver and adenomatous hyperplasia of bile duct were obviously decreased in the *B.s*-CotC-CsPmy-treated group after the challenging infection.

The SDS-PAGE showed that there was a fusion protein band on the theoretical site (108.8 kDa) in *B.s*-CotC-CsPmy lane (Fig. 3a). The immunoblotting assay (Fig. 3b) together with immunofluorescence analysis (Fig. 4) further confirmed the protein expression and localization on the surface of recombinant spores. Furthermore, when recombinant spores were preserved under room temperature for three months, no obvious degradation of the fusion protein was presented (Fig. 3c). These results showed that the fusion protein (CotC-CsPmy) was abundantly and stably expressed on the surface of recombinant spore and indicated the perfect thermostability of fusion protein. Our results are in concordance with previous reports about the thermostability of fusion protein on spores [22]. These features facilitate the storage and transport of the recombinant spores [45].

When the recombinant spores were intraperitoneally injected into mice, the CsPmy specific antibody was significantly increased in week 2 and continually ascended to week 6 (Fig. 5a). These results were in accordance with that of rCsPmy purified from *E. coli* reported in a previous study [33]. It demonstrated that CsPmy expressed on the spore surface preserved strong immunogenicity. Additionally, both IgG1 and IgG2a levels increased from week 2 to week 6 post-immunization (Fig 5b), indicating that combined Th1/Th2 immune responses were successfully provoked by *B.s*-CotC-CsPmy recombinant spores. The increased IgG isotype also manifested that combined cellular immunity and humoral immunity had been successfully induced by intraperitoneal injection of *B.s*-CotC-CsPmy spores.

While ingested by definitive hosts, metacercariae excyst in the duodenum. Larvae then migrate *via* the hepatopancreatic ampulla to the intrahepatic bile ducts in a short time and finally develop into adult worms. The juvenile flukes attach themselves to the bile-duct epithelium using their suckers so that they can travel against the bile flow along the biliary tree [2, 46]. During the invasion and parasitism process, the intestinal mucus and bile duct play vital roles in the interaction between the parasite and the host. Thus, for this kind of non-intravascular parasite, local mucosal immune responses on the intestinal and bile duct mucosa may be more important against *C. sinensis* infection. *Bacillus subtilis* spores have been widely employed as an antigen presenting vehicle for oral vaccines because of its safe, stable and adjuvant effect [13, 17, 45]. It has been demonstrated that spores could adhere to and be taken up by M cells on intestinal mucosa, then interact with macrophages, dendritic cells, or B cells in Peyer’s patches before being transported to the efferent lymph nodes, and finally induce specific antibodies secretion [37, 47]. Hence, we supposed that oral immunization with recombinant spores displaying CsPmy as a promising molecule on its surface could be an effective and needle-free method for prevention of *C. sinensis* infection.

As expected, after intragastrically administrated with recombinant spores, the specific IgG level in serum and in intestinal mucus of mice in the *B.s*-CotC-CsPmy group increased rapidly two weeks after the first immunization (Fig. 5c) and kept on rising in week 4 and week 6, which demonstrated that systemic immune response was successfully evoked. Both the IgG1 and IgG2a levels were significantly boosted from week 2 to week 6 (Fig. 5d), denoting that, as happened in intraperitoneally immunized mice, a combined Th1/Th2 immune response was aroused by ntragastrically administrated recombinant spores.

Compared with intraperitoneally immunized groups, the IgG in intestinal mucus and sIgA levels in intestinal mucus, feces and bile were higher and rose much earlier in intragastrically immunized mice (Fig. 6b, c, f-h). There was no difference in the sIgA level of bile in intraperitoneally immunized mice among groups of PBS, *B.s*-CotC, and *B.s*-CotC-CsPmy (Fig. 6d). These results indicate that oral immunization with spores elicited a stronger mucosal immune response in the intestinal mucosa and bile ducts. As reported, dominant antibodies exist in intestinal mucus, which serve as the first line of defense against pathogens including parasites [48, 49]. These play a crucial role in inhibiting the motility and adherence of pathogens in the intestinal mucus [49]. Studies have confirmed that specific sIgA to intestinal pathogens is also present in bile juice [50]. sIgA in bile juice was considered to play a more important role than in the intestine, given that the bile antibodies are in a more intimate contact with *C. sinensis* worms [10]. Our results show that sIgA in intestinal mucus of mice from the *B.s*-CotC-CsPmy group was obviously higher than that in control groups (Fig. 6b, f). In addition, the sIgA level of bile in the *B.s*-CotC-CsPmy group was also significantly accelerated in weeks 4 and 6 (Fig. 6h). This might be mostly due to large numbers of IgA-secreting cells being detected in duodenum, jejunum, ileum in the *B. subtilis* spores treated groups, especially in the *B.s*-CotC-CsPmy group (Fig. 7). These results indicated that recombinant spores endured the complex environment in the gastrointestinal tract and triggered both systemic humoral and local mucosal immune responses after oral administration.

*Bacillus subtilis* spores were used as food additives as no toxicity or virulence was observed for both *in vitro* and *in vivo* assessment [17]. GOT and GPT are the most sensitive and commonly employed indices for hepatocyte damage evaluation, as they may release into serum from the cytoplasm of the hepatic cell and resulting in an apparent increase in serum while hepatocyte was injured [51]. In our experiments, the activities of GOT and GPT were analyzed and no significant difference was detected in spore-treated groups and the control group (Fig. 8n). It manifested that there were no side effects on the liver function of mice orally administrated with recombinant spores. Additionally, no inflammation was observed in intestinal tissues of mice in each group by H&E staining (Fig. 8h-m), and the result was in accordance with those reported before [6, 21].

Intestinal bacteria develop and regulate the host immune system and the immune system, in turn, affects the composition of the intestinal microbiome [52]. To investigate whether intake of *B. subtilis* spores would change the composition of gut microbiota and break the gut homeostasis of mice, high-throughput sequencing combined with bioinformatics was used to decipher the impacts of spore on the structure of gut microbiota in spore-treated mice in detail. Results showed no significant difference in the OTUs numbers of the PBS group and the spores-treated group (Fig. 9b). At the phylum level, the dominant bacterial phyla were Bacteroidetes, Firmicutes and Proteobacteria covering more than 95% relative abundance in both groups (Fig. 9c). No significant difference in the indices (Chao1, ACE, Shannon, Simpson) between the spores-treated and control group was observed (Fig. 9d). These results illustrated that the bacterial richness and diversity of gut microbial community were not changed after the oral immunization program and the oral dosage of recombinant spores used was safe to the intestinal microflora.

The parasitism of *C. sinensis* in the liver could prompt collagen deposition or lead to adenomatous hyperplasia of the bile duct [2]. Our results showed that compared with the control group, collagen deposition in liver reflected by Ishak score and adenomatous hyperplasia of the bile duct in spore immunized (orally and intraperitoneally) groups were much less serious than in the control group (Fig. 8a). Paramyosin, present in numerous invertebrates including helminths, is a muscle protein that plays multifunctional roles in host-parasite relationships [25, 53]. The less expressed histopathological changes in *B.s*-CotC-CsPmy-immunized mice might be due not only to the lower quantity of *C. sinensis* eggs or adult worms but also to function inhibition of CsPmy by local or humoral anti-CsPmy antibodies including IgG and IgA. The exact mechanisms involved require further studies.

In recent years, some antigens from *C. sinensis* (including components of excretory-secretory antigens, tegumental proteins and enzymes related to metabolism) have been explored for their vaccine efficacy on rat models. The immunization of plasmids, containing genes encoding cysteine proteinase (CsCP), fatty acid-binding protein (CsFABP) and enolase (CsENO), elicited worm reduction rates of 31.50, 40.90 and 37.42%, respectively, in a rat model [2]. Oral immunization of *B. subtilis* spores expressing a 22.3 kDa tegumental protein of *C. sinensis* (CsTP22.3) and CsENO was also investigated in rat models. The worm reduction rates were between 44.70–60.07% with the egg reduction rates between 30.4–80.67% [22, 37].

In our study, the data for worm burden of immunized mice are not given, as the intrahepatic bile ducts of mice were so tiny that we failed to separate adult worms from the liver as happened before [6]. The egg reduction rate elicited by intraperitoneal (51.2%) and oral (48.30%) immunization with *B.s*-CotC-CsPmy spores was, however, higher than that elicited by the oral immunization with spores expressed with CsTP22.3 (30.4%), though it was lower than that elicited by PEB03-CotC-ENO spores (80.67%) [22, 37]. Additionally, the egg reduction rate elicited by *B. subtilis* spores displaying leucine aminopeptidase 2 (CsLap2) and cysteine protease (CsCP) were unknown [6, 21], so we could not compare the protective effect of B.s-CotC-CsPmy spores with that of CsLap2 and CsCP spores. It is worth comparing the protective effects elicited by different spores displaying mentioned antigens or by spores expressing the fusion gene of these antigens in future. Additionally, the protective effect elicited by intragastric immunization (48.30%) was slightly lower than that induced by intraperitoneal immunization (51.2%) without statistically significant difference (Table 1). It might be because the injection of spores elicited a stronger non-specific immune reaction than oral immunization, such as lysozyme and complement system, which might also take part in the elimination of *C. sinensis* in mice [23]. However, the mechanism involved deserves further exploration.

The different protective effects reflected by egg reduction rates might be related to the distinct function and immune character of different antigens. Immune response aroused by one antigen alone may not enough for the prevention of complex parasite. Therefore, we speculated that combined immunization with individual spores expressing antigens with different functions or spores with fusion peptide/protein might bring possibly better protective effects which deserve further investigation.

## Conclusions

CsPmy was successfully and abundantly expressed on the surface of *B. subtilis* spores with good thermostability, and preserved excellent immunogenicity. Both the systemic and local specific immune responses were provoked in mice after immunization with recombinant spores. Though it induced a stronger mucosal immune response, oral immunization elicited similar protective efficacy with intraperitoneal immunization. The egg reduction rate of intrgastic and intraperitoneal immunization was 48.3 and 51.2%, respectively. There was also no significant difference in the Ishak fibrosis scores of liver from mice treated with oral immunization and intraperitoneal immunization after challenging infection. Oral administration with B.s-CotC-CsPmy spores had no side-effect on liver function or intestinal histology and did not change the composition of gut microbiota, suggesting that oral immunization with *B. subtilis* spores expressing CsPmy on the surface may be a more convinient, safe and needle-free vaccination strategy against clonorchiasis.

## Abbreviations

B. s.: *Bacillus subtilis*
Chao1: Chao1 richness estimator
CotC: coat protein C, one of the coat proteins of *B. subtilis* spore
CsPmy: paramyosin of *C. sinensis*
EPG: eggs per gram faeces
GOT/AST: glutamic oxaloacetic transaminase/aspartate aminotransferase
GPT/ALT: glutamic pyruvic transaminase/alanine aminotransferase
OTUs: operational taxonomic units
rCsPmy: recombinant CsPmy protein
